# The effect of inlet and outlet boundary conditions in image-based CFD modeling of aortic flow

**DOI:** 10.1186/s12938-018-0497-1

**Published:** 2018-05-30

**Authors:** Sudharsan Madhavan, Erica M. Cherry Kemmerling

**Affiliations:** Department of Mechanical Engineering, Tufts University, 200 College Avenue, Medford, MA 02155 USA

**Keywords:** Inlet boundary conditions, Womersley, Windkessel, Outlet boundary conditions

## Abstract

**Background:**

Computational modeling of cardiovascular flow is a growing and useful field, but such simulations usually require the researcher to guess the flow’s inlet and outlet conditions since they are difficult and expensive to measure. It is critical to determine the amount of uncertainty introduced by these assumptions in order to evaluate the degree to which cardiovascular flow simulations are accurate. Our work begins to address this question by examining the sensitivity of flow to several different assumed velocity inlet and outlet conditions in a patient-specific aorta model.

**Methods:**

We examined the differences between plug flow, parabolic flow, linear shear flows, skewed cubic flow profiles, and Womersley flow at the inlet. Only the shape of the inlet velocity profile was varied—all other parameters were identical among these simulations. Secondary flow in the form of a counter-rotating pair of vortices was also added to parabolic axial flow to study its effect on the solution. In addition, we examined the differences between two-element Windkessel, three element Windkessel and the outflow boundary conditions. In these simulations, only the outlet boundary condition was varied.

**Results:**

The results show axial and in-plane velocities are considerably different close to the inlet for the cases with different inlet velocity profile shapes. However, the solutions are qualitatively similar beyond 1.75*D*, where *D* is the inlet diameter. This trend is also observed in other quantities such as pressure and wall shear stress. Normalized root-mean-square deviation, a measure of axial velocity magnitude differences between the different cases, generally decreases along the streamwise coordinate. The linear shear inlet velocity boundary condition and plug velocity boundary condition solution exhibit the highest time-averaged wall shear stress, approximately $8\%$ higher than the parabolic inlet velocity boundary condition. Upstream of 1*D* from the inlet, adding secondary flow has a significant impact on temporal wall shear stress distributions. This is especially observable during diastole, when integrated wall shear stress magnitude varies about $26\%$ between simulations with and without secondary flow. The results from the outlet boundary condition study show the Windkessel models differ from the outflow boundary condition by as much as $18\%$ in terms of time-averaged wall shear stress. Furthermore, normalized root-mean-square deviation of axial velocity magnitude, a measure of deviation between Windkessel and the outflow boundary condition, increases along the streamwise coordinate indicating larger variations near outlets.

**Conclusion:**

It was found that the selection of inlet velocity conditions significantly affects only the flow region close to the inlet of the aorta. Beyond two diameters distal to the inlet, differences in flow solution are small. Although additional studies must be performed to verify this result, the data suggest that it is important to use patient-specific inlet conditions primarily if the researcher is concerned with the details of the flow very close to the inlet. Similarly, the selection of outlet conditions significantly affects the flow in the vicinity of the outlets. Upstream of five diameters proximal to the outlet, deviations between the outlet boundary conditions examined are insignificant. Although the inlet and outlet conditions only affect the flow significantly in their respective neighborhoods, our study indicates that outlet conditions influence a larger percentage of the solution domain.

## Background

Cardiovascular computational fluid dynamics (CFD) models have the ability to aid physicians in non-invasive diagnostic decision making, and over the past decade, commercial, patient-specific modeling has become more common owing to numerous advancements in computing speed [1], medical image acquisition, and 3D data processing and visualization techniques [2–5].

Cardiovascular diseases (CVDs) are the leading cause of death globally [6], with the most common conditions including coronary artery disease (CAD), stroke, heart failure, rheumatic heart disease, heart arrhythmia, aortic aneurysms, and thromboembolic diseases [6, 7]. CAD and stroke account for about $77\%$ of CVD deaths [6], but many other conditions contribute to impairment or decreased quality of life of the patient. As a means to diagnosing and understanding these conditions, commercial, patient-specific modeling of CVDs has become more common in recent years. For instance, HeartFlow, Inc., Redwood City, California has developed a non-invasive CFD-based tool to identify lesions causing ischemia [8, 9]. Another application of cardiovascular CFD is designing new surgical techniques and implantable medical devices [10, 11]. Procedures and devices have traditionally been validated via clinical trials, animal tests, and evaluation of patients post-surgery. Cardiovascular modeling is now increasingly aiding these developments [11–18]. For example, [10] designed a ‘virtual surgery’ for pediatric surgeons based on patient-specific images. Their framework also computed post-operative hemodynamics based on the virtual surgery, thereby aiding surgeons in surgical planning. Furthermore, hemodynamic alterations are known to be a significant cause of ischemic disease progression [19]. Owing to these uses and other promising applications, there is a substantial need for accurate modeling of cardiovascular flows.

Unfortunately, much of the information required to perform accurate cardiovascular CFD is usually unavailable due to the difficulty of making in vivo flow measurements on live patients. Consequently, in order to formulate a well-posed problem, most researchers must guess parameters such as flow boundary conditions, vessel wall properties, and sometimes even geometric vessel parameters if patient imaging is not of sufficient quality. It has been shown that these factors and others can significantly alter the flow solution [20–25]. For example, [25, 26] performed a numerical study to quantify the sensitivity of wall shear stress fields in the carotid bifurcation to geometric and secondary flow perturbations. They found that small geometric variations could significantly affect the flow solution. Sankaran et al. [27] quantified uncertainties due to geometry, boundary conditions, and blood viscosity in coronary blood flow simulations using a stochastic collocation method [28]. They concluded that solutions from modeling were most sensitive to variations in minimum lumen diameter. Sankaran et al. [29] developed a reduced-order model based on a machine learning approach to quantify uncertainties due to geometric variations. They found that larger arteries with significant stenosis were most sensitive to geometric variations. Liu et al. [19] modeled a patient-specific circle of Willis coupled with a zero-dimensional lumped parameter boundary condition. They determined that the accuracy and consistency of their method were improved relative to a resistance-based boundary condition. Steinman et al. [22] was a collective study by 25 research groups to predict the variability of pressure drop in a giant aneurysm model with a proximal stenosis. Various research groups performed CFD analysis with the same lumen geometry, flow rates, and fluid properties. However, the researchers were free to choose their own numerical methods, discretization, and solution strategies. They concluded that pressure could be predicted with reasonable accuracy by CFD in the giant aneurysm model but transitional patterns and derived quantities varied widely. Liu et al. [30] developed a new methodology for functional assessment of stenotic carotid arteries. Their methodology based on thresholding pressure gradient successfully delineated severe stenosis from mild-moderate ones. Xiong et al. [31] investigated the effect of blood pressure variability on carotid atherosclerotic plaques. They determined that beat-to-beat blood pressure variability could severely exacerbate long-term outcomes of atherosclerosis. Wong et al. [32] studied the effect of fluid structure interaction on carotid bifurcation models with varying degrees of atherosclerosis. They concluded that wall shear stress and geometric deformation are significantly influenced by the severity of the disease. Liu et al. [33] simulated fluid structure interaction of blood flow and elastic arteries with eccentric stenotic plaques. They showed that wall shear stress, pressure drop and von Mises stress were positively correlated with the degree of vessel occlusion via plaques. Pekkan et al. [23] examined variations between solutions from a first-order accurate commercial software and a second-order accurate in-house flow solver. Only the second-order methods could accurately match the three-dimensional flow features found in an experimental model. Recent studies [20, 21] showed the effect of mesh resolution on patient-specific models and concluded that a typical mesh resolution in comparison to a higher mesh resolution resulted in pronounced underestimation of quantities such as wall shear stress and oscillatory shear index. They also showed that higher resolution meshes were able to capture flow instabilities.

Since cardiovascular CFD simulations are used to make critical decisions in diagnosis [30], surgical planning [10], and medical device designs [12, 13, 15], it is essential to verify that the assumptions made by the researcher do not negatively impact the fidelity of the solution. In this paper, we focus on the impact on flow solution of assumed inlet velocity boundary conditions in the human aorta. Some have argued that researchers concerned about the choice of inlet conditions should merely extend the size of the simulation domain so the flow is fully-developed by the time it reaches the point of interest. However, this is rarely a realistic solution since real arteries are poorly approximated by long, straight tubes, thus the flow is never truly fully-developed within the body. Furthermore, it is often prohibitively complex to add realistic upstream sections of the vasculature, as in the case of the aorta, which is immediately distal to the heart.

The aorta is of particular interest not only due to its position proximal to all other arteries, but also because invasive and non-invasive experimental measurements on the aortic arches of animals and humans have reported wide variations in the shape of the velocity profile, including flat [34], skewed [35], and highly patient-specific [36]. Consequently, in cases where patient-specific profiles are unavailable, the optimal profile shape to assume is not clear, and researchers have made many different choices [37–44]. To our knowledge, it is thus far undetermined to what extent the researcher’s choice of aortic inlet boundary condition changes the solution, or how far distal to the inlet the flow is significantly affected by the choice of inlet condition. In addition, it is not always clear how the choice of outlet boundary condition affects the flow solution; most researchers choose between an outflow outlet condition, in which flowrate is specified at each outlet, and a Windkessel model, in which distal resistances and capacitances are modeled [45–49]. It is critical to answer these questions to determine the extent to which the hundreds of published studies with non-patient-specific inlet and outlet conditions are accurate. In the current study, we begin to address these issues by simulating aortic flow with a variety of idealized inlet and outlet conditions. At the inlet, we examine plug flow, parabolic flow with and without secondary flow, linear shear flows, skewed cubic profiles, and Womersley flow. At the outlet, we study the two-element and three-element Windkessel models and compare them with specified mass flow rate and zero diffusion flux  (*ANSYS® Academic Research [Fluent], release 16.2, outflow boundary conditions, ANSYS, Inc.*). The overall goal is to quantify the differences in flow solution caused by choice of inlet and outlet conditions for the purposes of evaluating the impact of assumed boundary conditions on previously-published aortic flow studies.

## Methods

**Fig. 1:**
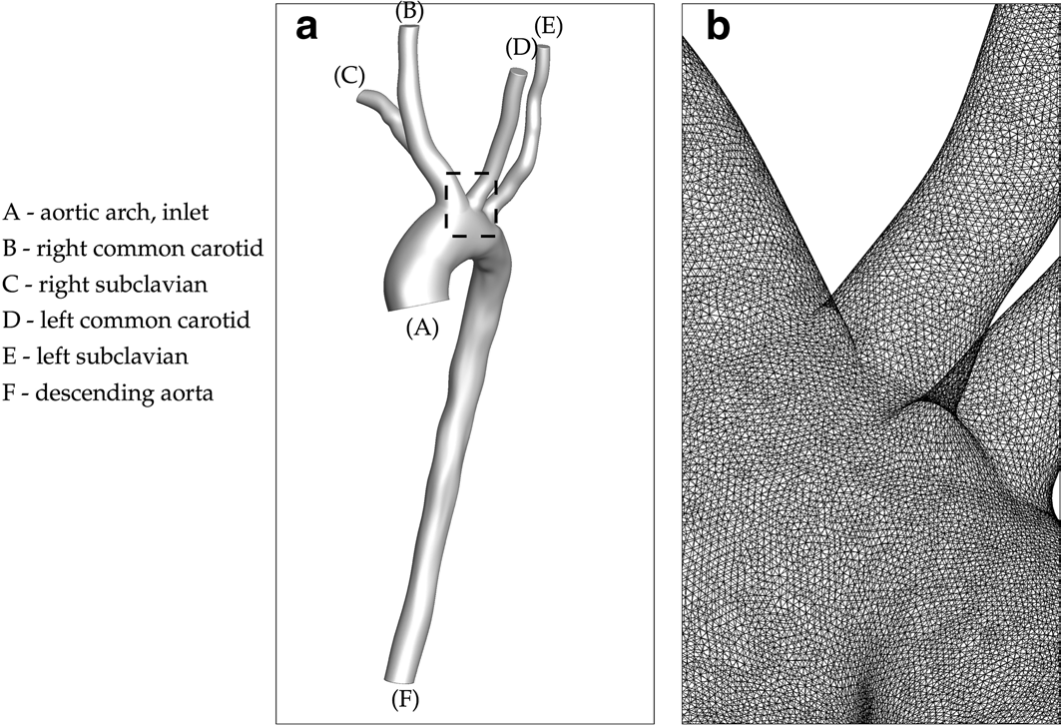
**a** Image-based model of a subject-specific vasculature; **b** a magnified view of the computational mesh of the subject-specific vasculature

An image-based model of a patient-specific aorta of a healthy adult including the brachiocephalic trunk, common carotid arteries, and subclavian arteries was obtained through a personal correspondence (A. Marsden, personal communication, January 11, 2016). A perspective view of the model is shown in Fig. 1a.

**Fig. 2:**
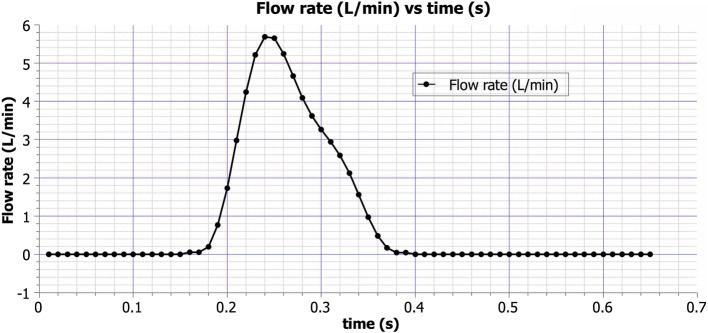
Aortic blood flow rate waveform $\left( \frac{L}{min}\right)$ versus time (*s*) [68]

**Fig. 3:**
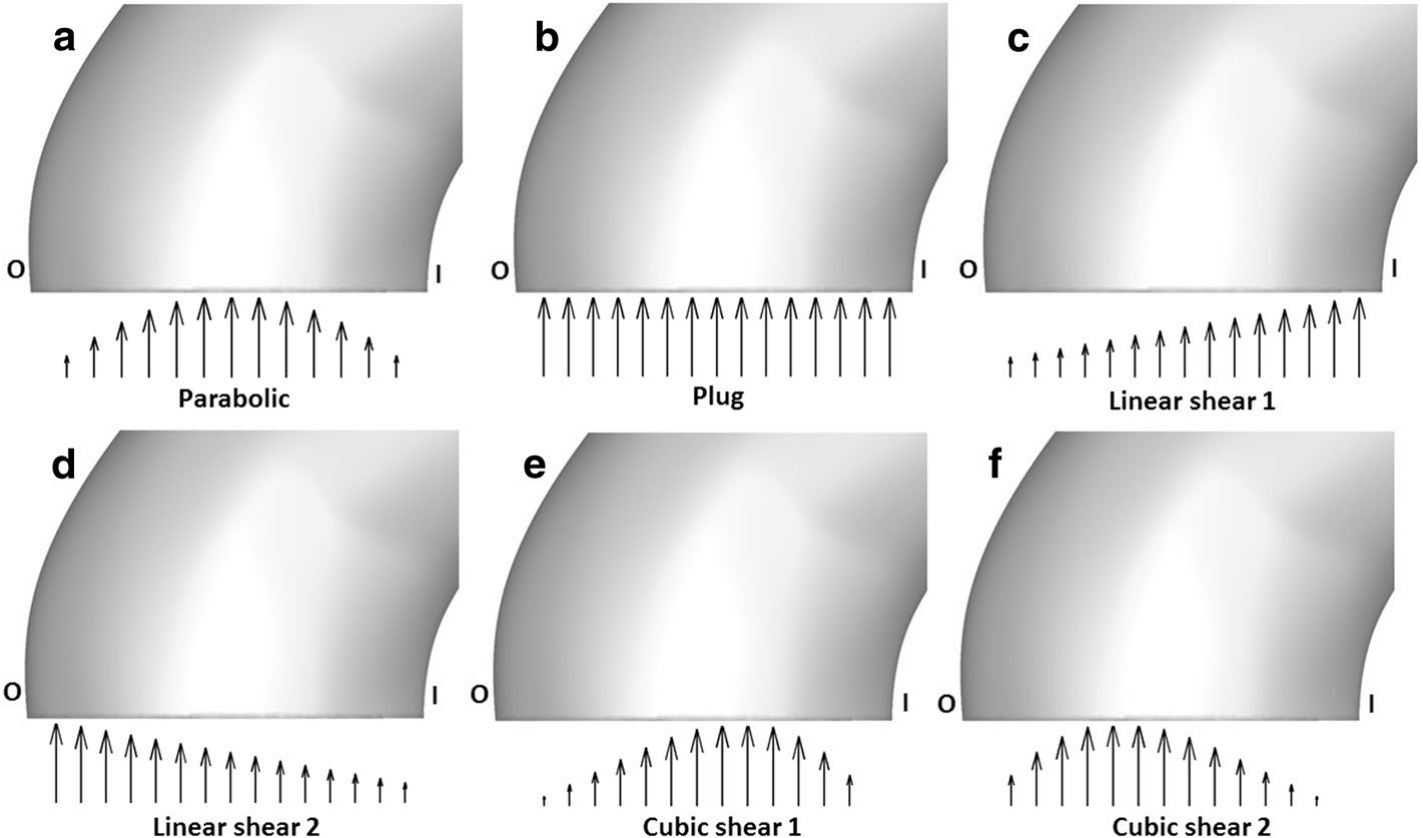
Select inlet velocity conditions; **a** parabolic, **b** plug, **c** linear shear 1, **d** linear shear 2, **e** cubic shear 1, **f** cubic shear 2; ’I’ and ’O’ indicate the inner and outer curve of the aortic arch, respectively

**Fig. 4:**
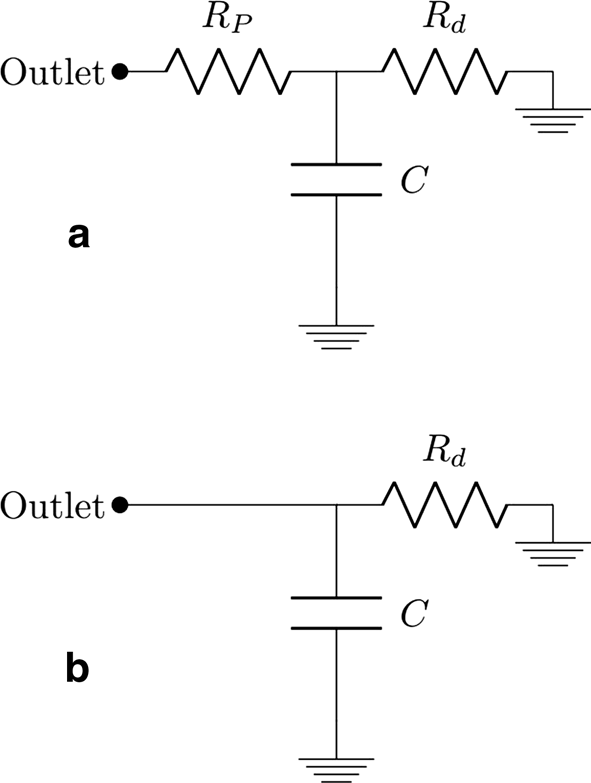
A schematic of the Windkessel models **a** three-element Windkessel model, **b** two-element Windkessel model

The commercial CFD software package ANSYS Fluent (*ANSYS® Academic Research [Fluent], release 16.2*) was used for our analysis. The built-in ANSYS meshing tool was employed to discretize the patient-specific geometry using tetrahedral grid elements. We solved the incompressible 3D Navier–Stokes equations shown in Eq. 1 using a finite volume discretization. While the pressure was computed using a second order discretization, the momentum was determined employing a second order upwind scheme. Pressure and velocity were coupled following the Semi-Implicit Method for Pressure Linked Equations (SIMPLE) algorithm [50], (*ANSYS® Academic Research [Fluent], release 16.2, 25.9.1, Choosing the Pressure–Velocity Coupling Method, ANSYS, Inc.)*. We required the scaled residual criteria defined in  (*ANSYS® Academic Research [Fluent], release 16.2, 25.18.1, Monitoring Residuals, ANSYS, Inc.*) to be less than $10^{-3}$ for convergence. This corresponds conceptually to a percentage error of less than $0.1\%$ in the solution to the Navier–Stokes equations and is consistent with the recommendation from (*ANSYS® Academic Research [Fluent], release 16.2, ANSYS, Inc.*). Blood was modeled as a Newtonian fluid with a density of $1060\,{kg}/{m^{3}}$ [51] and a viscosity of 0.004 Pa  s [52]. Although Newtonian models consistently underestimate significant physiological factors such as wall shear stress, the qualitative patterns have been shown to be similar to those predicted by non-Newtonian models [53–58]. In particular, the average difference in wall shear stress between Newtonian and non-Newtonian models, as demonstrated by [57, 58], is about $10\%$. We also assumed the vessel walls to be rigid, which has been shown to overestimate quantities such as instantaneous wall shear stress. However, time-averaged wall shear stress has been shown to vary by only about $4.5\%$ [59–61]. Moreover, this work is an attempt to study the effect of varying inlet boundary conditions along with the most commonly assumed parameters in cardiovascular simulations [62–66], rather than to perform an optimally realistic simulation of aortic flow.

1 $$\begin{aligned} \frac{\partial {u}_{i}}{\partial t}+u_j \frac{\partial {u}_{i}}{\partial x_j}&=-\frac{1}{\rho } \frac{\partial p}{\partial x_i}+\nu \frac{\partial ^2 u_i}{\partial x_j \partial x_j}\nonumber \\ \frac{\partial {u}_{j}}{\partial x_j}&= 0 \end{aligned}$$

In the present study, 6,484,130 tetrahedral elements were used to discretize the geometry with a minimum element size of $6.98 \times 10^{-5}$ m and a maximum element size of $2.52 \times 10^{-4}$ m. Doubling the number of elements contributed to only about $1.8\%$ root-mean-square (RMS) differences in the velocity magnitude. A zoomed-in section of the grid is shown in Fig. 1b. Temporally, we employed a first order implicit scheme with a time step of 0.01 s. This scheme was found to be both stable and efficient with our model in comparison to the other options.

### Inlet sensitivity studies

In the first part of this study, the sensitivity of flow solutions to velocity inlet conditions was investigated. For these simulations, a zero diffusion flux for all flow variables at the outlets and an overall outlet flow rate were employed to impose specified $\%$ mass flow splits (*ANSYS® Academic Research [Fluent], release 16.2, 7.3.10, outflow boundary conditions, ANSYS, Inc.*). The average outflow rates were obtained from [45, 67].

The average outlet flow rates in the daughter vessels are shown in Table 1. Inlet boundary conditions in the model were set up using user-defined functions (UDFs). An external code (in C++) was written to generate custom inlet velocity boundary conditions. The total flow rate vs. time waveform, shown in Fig. 2, was adapted from [68]. Eighth order Fourier decomposition of the aforesaid waveform was used in the current study. For the different simulation cases, the shape of the inlet velocity profile was varied without changing the flow rate. Plug flow, parabolic flow, linear shear flows, skewed cubic flow profiles, and Womersley flow were examined. A schematic of all the primary flow inlet conditions examined except the Womersley condition is shown in Fig. 3. Womersley flow with an identical flow rate was modeled following the formulations of [69]. In addition to the aforementioned conditions, a parabolic primary inlet flow with a counter-rotating vortex pair secondary flow was simulated. The numerical formulation of this secondary flow is described by Eqs. 2 and 3. The mean secondary flow speed was $24\%$ of the mean primary flow speed, as reported in [70], during the systolic periods. Realistic secondary flow in some vessels can be modeled by adding a simple proximal geometry extension, as is done for coronary arteries in [25]. However, such a model is not accurate for the aorta due to the complex in vivo upstream conditions caused by the beating heart and the aortic valve. In the current study, the secondary flow specified by Eqs. 2 and 3 was selected not because it accurately represented flow in an in vivo aorta, but because it aided evaluation of the effect on the flow of an arbitrary secondary flow of reasonable strength and shape [71–74]. The effect of secondary flow was studied on the parabolic primary velocity profile since it is the most commonly assumed primary velocity profile shape in cardiovascular simulations.

**Table 1:** Outflow boundary conditions

Vessel	Outlet flow rate
B-right common carotid	9.8
C-right subclavian	9.5
D-left common carotid	5.2
E-left subclavian	6.4
F-descending aorta	69.1

**Table 2:** Parameters for the Windkessel outlet boundary conditions, adapted from [77]

Vessel	***R***_***p***_ (dynes s/cm^5^)	C (cm^5^/dynes)	***R***_***d***_ (dynes s/cm^5^)
Right common carotid	1180	7.70E−5	18,400
Right subclavian	1040	8.74E−5	16,300
Left common carotid	1180	7.70E−5	18,400
Left subclavian	970	9.34E−5	15,200
Descending aorta	188	4.82E−4	2950

Note $R_p$ was needed only for the three-element outlet condition, but *C* and $R_d$ were used for both the three- and two-element conditions

2 $$\begin{aligned} \overrightarrow{V}~=~K(t)~\cdot ~\left[ \frac{-(y-y_1)}{(r-r_1)^2}+\frac{(y-y_2)}{(r-r_2)^2} \right] \cdot \hat{\mathbf{j }} \end{aligned}$$

3 $$\begin{aligned} \overrightarrow{W}~=~K(t)~\cdot ~\left[ \frac{(x-x_1)}{(r-r_1)^2}-\frac{(x-x_2)}{(r-r_2)^2} \right] \cdot \hat{\mathbf{k }} \end{aligned}$$

In Eqs. 2 and 3, $\overrightarrow{V}$ and $\overrightarrow{W}$ are velocity vectors perpendicular to the axial velocity vector, $\overrightarrow{U}$; $(x_1,~y_1)$ and $(x_2,~y_2)$ are the coordinates of the centers of vortices; $r_1~=~\sqrt{x_1^2+y_1^2}$ and $r_2~=~\sqrt{x_2^2+y_2^2}$. $\overrightarrow{V}$ and $\overrightarrow{W}$ were set to $\overrightarrow{0}$ at the vortices’ centers ($<15\%$ of vessel radius) to suppress the blowup of velocity components. *K*(*t*) was chosen to ensure that the mean secondary flow speed was $24\%$ of the mean primary flow speed as reported in [70].

### Outlet sensitivity studies

In the second part of this study, we examined the sensitivity of the flow to the choice of outlet boundary conditions, focusing on those most commonly assumed: the outflow condition and the three-element (RCR) and two-element (RC) Windkessel models [75–77]. For these simulations, a parabolic inlet velocity condition was prescribed and there was no secondary flow at the inlet. The Windkessel model was invented in 1899 [78] and later adapted to model transient outflow boundary conditions in [75]. The three-element Windkessel model is an electric-circuit analogue consisting of a proximal resistance, $R_p$ in series with a parallel network of a capacitor, *C*, and a distal resistance, $R_d$, as shown in Fig. 4a. The two-element model is identical to the three-element model except for the absence of proximal resistance, as shown in Fig. 4b. While the proximal resistance models the viscous resistance of the vasculature immediately downstream of the vessel, the distal resistance accounts for the resistance of the capillaries and the venous circulation. The capacitor is representative of the compliance of the downstream vessels. Assuming such an analogue yields us Eq. 4 [75, 79, 80]. The outlet pressure was then obtained using an implicit time discretization of Eq. 4 as described in [80].

4 $$\begin{aligned} \frac{\partial p}{\partial t} + \frac{p}{C R_d} = \frac{Q}{C} \left( 1+\frac{R_p}{R_d}\right) +R_p \frac{\partial Q}{\partial t} \end{aligned}$$

In Eq. 4, *p* represents the outlet pressure, and *Q* represents the flow rate through the vessel. Typically resistance and capacitance parameters for the Windkessel model are tuned to match the outlet flowrate from the in vivo model. However, since flowrates through the outlets were unavailable for this particular patient, these parameters were adapted from a similar aorta model [77]. Table 2 lists resistance and capacitance values used for the various daughter vessels.

For all simulations, flow was assumed to be laminar since the Reynolds number, $\mathbf {Re}_{D}$ based on the inlet aortic diameter, *D* was about 1700 at peak systole. The simulations were performed until the fifth cardiac cycle. Wall shear stress (*WSS*), pressure, and vorticity contours were examined from the fifth cardiac cycle. The centerline of the model was computed and data slices perpendicular to the centerline were extracted at streamwise coordinates that were multiples of the aortic root diameter, *D*. Time-averaged wall shear stress (*TAWSS*) and other time-averaged flow quantities were also computed by averaging over the fifth cardiac cycle. Results comparing the various inlet and outlet boundary conditions are presented in the following sections.

## Results and discussion

### Effect of the shape of the inlet axial velocity profile

This subsection discusses the influence of the axial velocity profile shape on the solution. These flows had no secondary flow at the inlet.

**Fig. 5:**
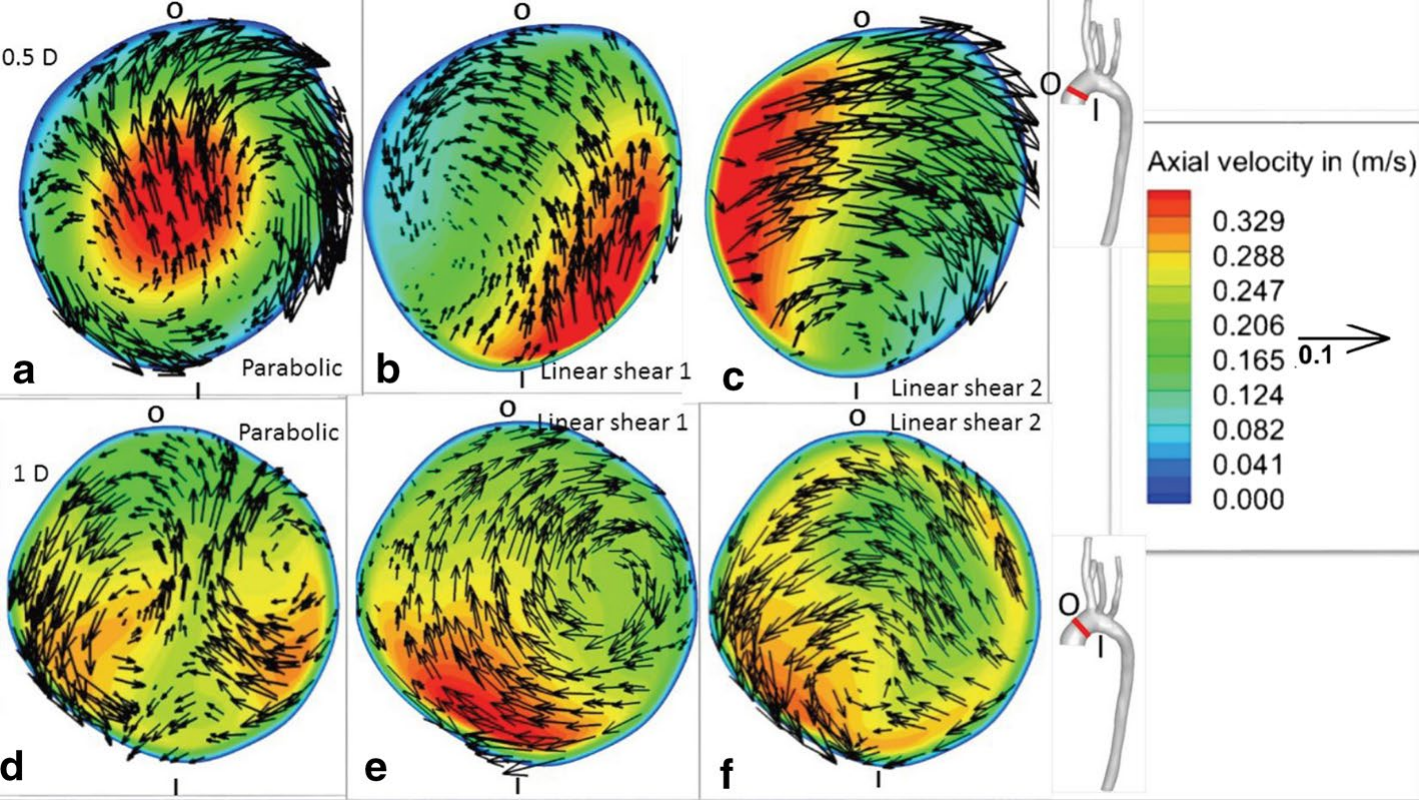
Axial velocity magnitude contours and tangential velocity vectors along planes normal to the centerline at streamwise coordinates of 0.5*D* and 1*D*, where D is the inlet diameter, during peak systole; **a**, **d** have parabolic velocity inlet conditions, **b**, **e** have linear shear velocity 1 inlet conditions, and **c**, **f** have linear shear velocity 2 inlet conditions. These simulations had no secondary flows at the inlets and all outlet boundary conditions were of the outflow variety

Data slices perpendicular to the centerline of the model were extracted at various locations along the aorta. Figure 5 shows data at streamwise coordinates of 0.5*D* and 1*D*, where ‘*D*’ is the diameter of the aorta’s inlet. Axial velocity magnitudes are depicted by contours. In-plane velocities are represented by the vectors in Fig. 5. Surfaces closer to the inner and the outer arch are denoted by the letters ‘I’ and ‘O’ respectively. The effect of inlet boundary conditions is more pronounced closer to the inlet of the vessel. For instance, the peak in axial velocity is approximately at the center of the cross-section for the parabolic inlet boundary condition, as shown in Fig. 5a. Similarly, the contours in Fig. 5b, c show marked similarities to their respective inlet conditions, linear shear flows 1 and 2. Owing to inertia, flow inside the curved vessel gets pushed towards the outer side of the arch, labeled ‘O’. This effect is apparent in the in-plane velocity vectors of the parabolic velocity inlet cross section in Fig. 5a. The counter-rotating vortex (CRV) pair, formed because of the aforesaid effect [81, 82], is retained at a streamwise position of 1*D* for the parabolic inlet boundary condition. In addition to the CRV pair, there is a smaller vortex closer to the inner arch, ‘I,’ for the parabolic inlet boundary condition. A counterclockwise rotating vortex is present in the flow with the linear shear 1 inlet condition. However, the linear shear 2 inlet has a clockwise rotating vortex, observed in Fig. 5c. For linear shear flow inlet boundary conditions, there is a change in the direction of rotation of the tangential velocity vectors with increasing streamwise coordinate. This effect can be observed by comparing Fig. 5b, e for the linear shear 1 inlet condition. A similar trend is also noticeable in Fig. 5c, f for the linear shear 2 inlet boundary condition. It is also notable that both the primary and the secondary in-plane flows look considerably different for the three boundary conditions illustrated in Fig. 5, but in all three cases, secondary flows are only a small percentage of the total flow velocity.

**Fig. 6:**
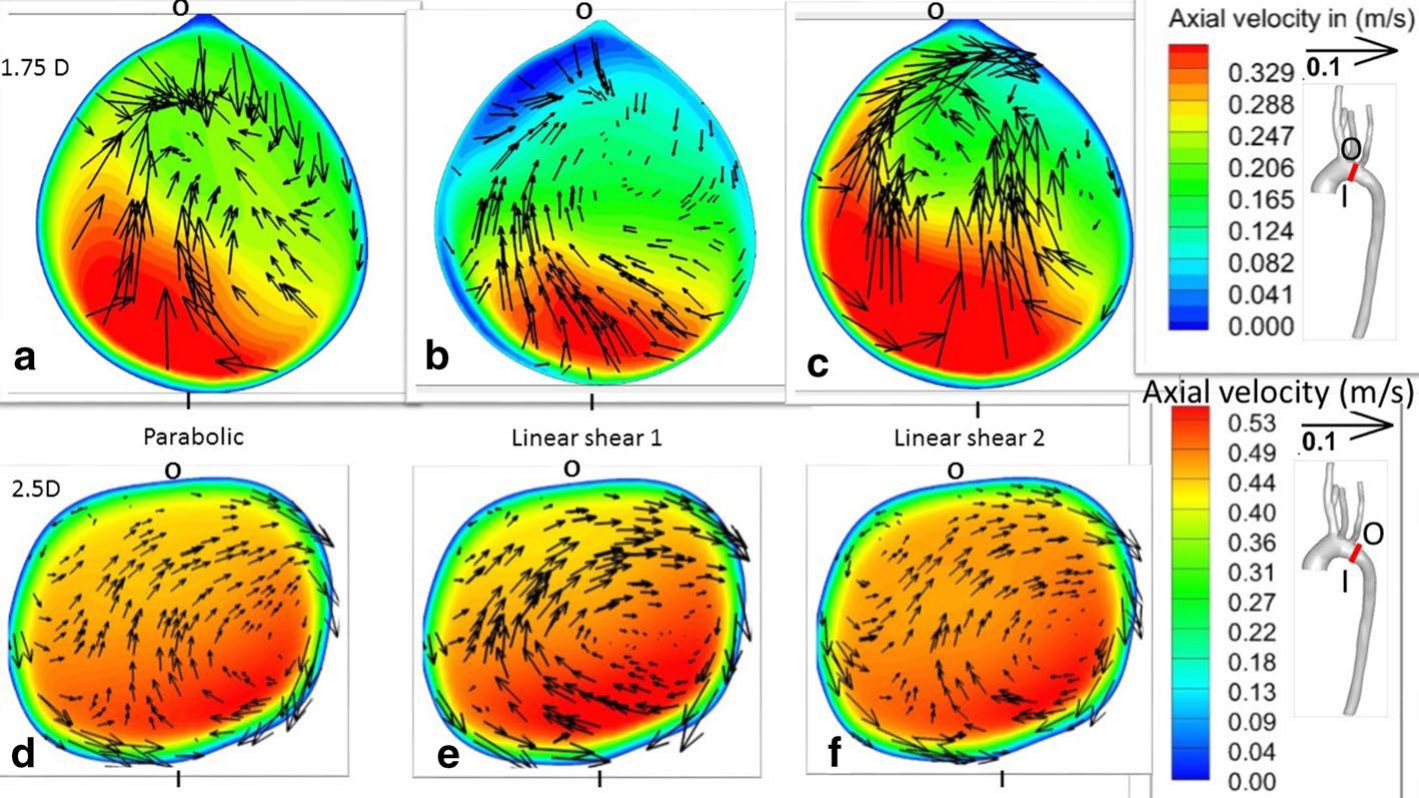
Axial velocity magnitude contours and in-plane velocity vectors along planes normal to the centerline at 1.75 and 2.5 inlet diameters downstream from the inlet during peak systole; **a**, **d** parabolic velocity inlet, **b**, **e** linear shear velocity 1 inlet, **c**, **f** linear shear velocity 2 inlet; note that the scales of the axial velocity contours are different for the two cross sections illustrated

Figure 6 shows data slices at streamwise distances of 1.75*D* and 2.5*D* from the inlet, where *D* is the inlet diameter, during peak systole. At these cross-sections, all boundary conditions shown yielded a clockwise-rotating secondary flow. Branching vessels have been shown to have a considerable effect on the secondary flow [40] so it is possible this was caused by the branching daughter vessels and the effect of the curvature of the vessel [40, 83, 84]. The velocity of the streamwise flow is skewed towards the inner wall of the vessel. This result agrees well with various other studies such as [40, 85–88], which have observed reversed and skewed flow along the inner wall of the vessel. Although a direct validation of our simulation cannot be performed due to lack of availability of patient velocity data, the qualitative features from our simulations match well with previous aortic flow studies as indicated above.

**Fig. 7:**
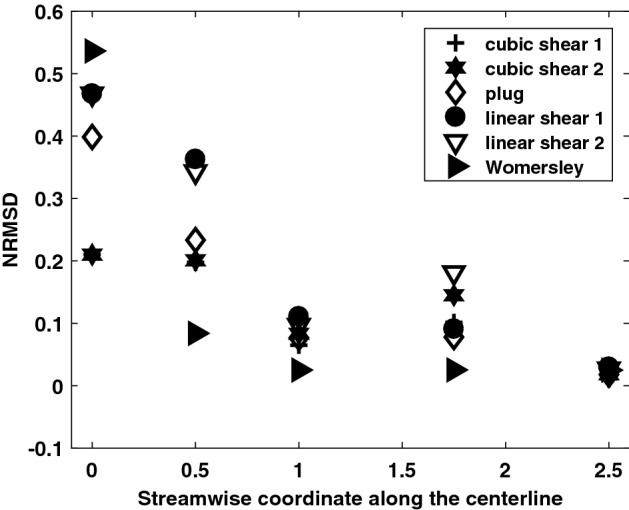
Normalized root-mean-square deviation (*NRMSD*) of axial velocity magnitude compared with parabolic inlet boundary conditions at various locations along the streamwise coordinate during peak systole; the streamwise coordinate is measure d in multiples of *D*, the vessel’s inlet diameter

There are a few minor differences between the three cases shown in Fig. 6, such as the shape of the peak in axial velocity contours and the direction of vectors in the secondary flow, especially at 1.75*D*. The differences in axial flow may be caused by a combination of the varying inlet velocity profiles and distortions to the secondary flow caused by the vessel’s curvature.

5 $$\begin{aligned} NRMSD~=~\frac{\sqrt{\frac{\sum \limits _{i=1}^{n} \left( \left( \overrightarrow{U}_{parabolic}\right) _i-\left( \overrightarrow{U}_{inlet~condition} \right) _i \right) ^2}{n}}}{\overline{\left( \left( \overrightarrow{U}_{parabolic} \right) _{i} \right) }} \end{aligned}$$

Figure 7 quantifies differences between various inlet boundary conditions and the parabolic inlet velocity boundary condition using normalized root-mean-square deviation (*NRMSD*) of axial velocity magnitude as described in Eq. 5, integrated over cross-sectional slices at the coordinates indicated. *NRMSD* generally decreases with increasing streamwise coordinate, although there is a slight increase at 1.75*D*. It is notable that *NRMSD* is within 0.03 at 2.5*D* for every inlet boundary condition examined. This is more than an order of magnitude smaller than its value at the inlet for most boundary conditions.

**Fig. 8:**
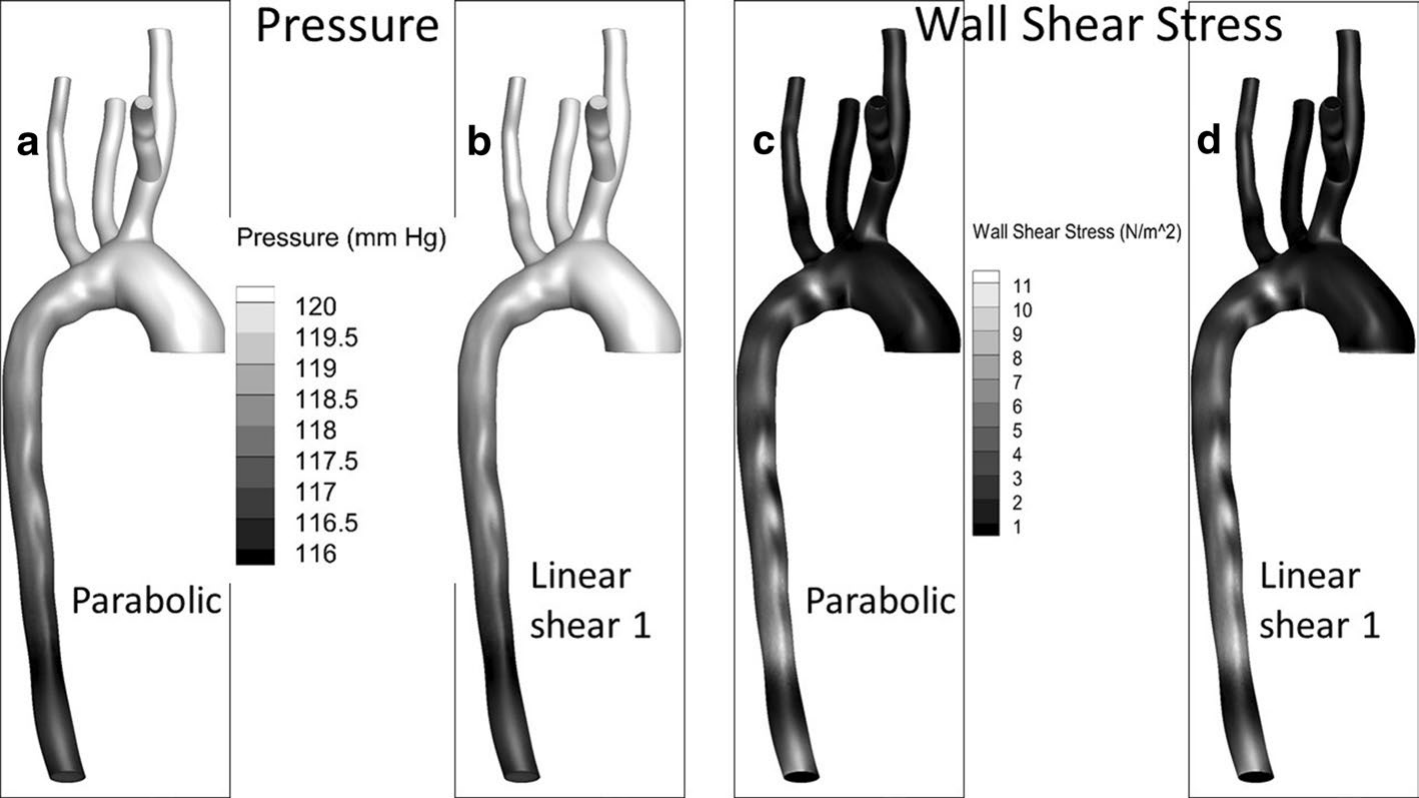
Pressure and wall shear stress (*WSS*) contours along the wall of the vessel during peak systole; **a**, **c** parabolic inlet condition, **b**, **d** linear shear 1 inlet condition

Figure 8 compares surface pressure and wall shear stress contours for two representative inlet velocity profiles: parabolic and linear shear 1. The two cases are very similar except for minor differences close to the inlet of the vessel. This was also typical for other inlet velocity profile cases not shown in the figure.

6 $$\begin{aligned} & {\text {Integrated wall shear stress \ (}} WSS {\text{)}}& \nonumber \\& = \left| \frac{100 \cdot \int _{wall} { (\tau _{w})_{inlet~condition}}{-(\tau _{w})_{parabolic} } ~dA}{ \int _{wall} (\tau _{w})_{parabolic} dA}\right| \end{aligned}$$

7 $$\begin{aligned} {\text {Time averaged wall shear stress \ (}}TAWSS{\text{ )}}&\nonumber \\&=\left| \frac{100 \cdot \int _{cardiac~cycle} \int _{wall} {(\tau _{w})_{inlet~condition}}{-(\tau _{w})_{parabolic} }~dA \cdot dt}{\int _{cardiac~cycle} \int _{wall} (\tau _{w})_{parabolic}~dA \cdot dt} \right| \end{aligned}$$

Table 3 shows differences between integrated wall shear stress of flows with different inlet conditions compared with the parabolic inlet condition, calculated using Eqs. 6 and 7. The spatial integrals in the aforementioned equations were computed following (*ANSYS® Academic Research [Fluent], release 16.2, 20.3, Surface Integration, ANSYS, Inc.*). Temporally, the integrals were calculated using a composite trapezoidal rule. The table contains comparisons for integrated wall shear stress at peak systole (Eq. 6) and time-averaged wall shear stress over a cardiac cycle (Eq. 7). Both of these parameters are integrated over the entire simulation domain. These differences are also quantified locally across the vessel wall up to 1*D* from the inlet. Linear shear flow 1 and plug flow exhibit the largest differences integrated over the entire domain, about $8\%$ in time-averaged wall shear stress. During peak systole these numbers are as high as $15\%$ for plug flow. However, in the first 1*D* from the inlet, linear shear flow 1 has the largest local variations, about $18\%$ in time-averaged wall shear stress and about $33\%$ in integrated wall shear stress during peak systole. It is also notable that the parabolic inlet condition has the lowest integrated wall shear stress and time-averaged wall shear stress among the inlet conditions examined.

**Table 3:** Differences in wall shear stress magnitudes compared with the parabolic inlet velocity boundary condition case as defined in Eqs. 6 and 7

Velocity boundary condition	Differences in *TAWSS* ($\%$)	Differences in *WSS* during peak systole ($\%$)	Differences in *TAWSS* up to 1*D* ($\%$)	Differences in *WSS* up to 1*D* during peak systole ($\%$)
Plug	8.72	15.69	7.21	31.48
Womersley	0.86	6.20	6.84	17.30
Linear shear flow 1	8.09	11.21	18.50	32.53
Linear shear flow 2	0.99	6.42	1.47	17.24
Cubic shear flow 1	5.08	0.63	12.73	2.67
Cubic shear flow 2	1.87	1.83	4.16	5.48

**Table 4:** Differences in wall shear stress magnitudes of the parabolic inlet velocity boundary condition cases with and without secondary flow

Integration domain	Differences in *TAWSS* ($\%$)	Differences in *WSS* during peak systole ($\%$)	Differences in *WSS* during diastole
Entire wall surface	3.58	0.19	5.35
Wall surface up to 1*D*	7.74	1.50	26.56

The secondary flow used is defined in Eqs. 2, 3

### Effect of adding secondary flow to the inlet

In this subsection, the effect of adding secondary flow to a parabolic axial inlet velocity profile is discussed. Only the parabolic axial flow is considered since it is the most commonly assumed inlet velocity profile shape in cardiovascular simulations.

Table 4 illustrates the variations in wall shear stress magnitudes between parabolic inlet flows with and without secondary flow at the inlet. Wall shear stress magnitude variations are significantly higher during diastole. Wall shear stress magnitudes vary the most near the inlet, but this phenomenon is also observed when wall shear stress is integrated over the entire domain.

**Table 5:** Differences in wall shear stress magnitudes between the three-element Windkessel (RCR), the two-element Windkessel (RC), and the prescribed percentage flow rate outlet (outflow) boundary conditions

WSS comparision	Differences in *TAWSS* ($\%$) up to 1*D* (%)	Differences in *WSS* up to 1*D* ($\%$) during peak systole	Differences in *TAWSS* ($\%$)	Differences in *WSS* ($\%$) during peak systole
RCR and outflow	0.3571	2.8515	18.2248	14.2861
RC and outflow	0.3544	2.8528	18.2758	14.3076
RCR and RC	0.0027	0.0013	0.0431	0.0250

All cases here had a parabolic inlet velocity profile and no secondary flow at the inlet

The magnitude of these differences must be interpreted in the context of other uncertainties in cardiovascular flow simulation. For example, in an image-based coronary arterial model examined by [25, 26], different models of blood rheology accounted for about $8\%$ variability in the solution, the effect of secondary inlet flow yielded $13\%$ variability, and geometric uncertainties resulted in $47\%$ variability in wall shear stress. It is notable that they generated secondary flow using an extension to their model with added curvature and helical pitch. Another study, [83], examined the effect of curvature and inlet velocity profile on a right coronary artery model. They concluded that inlet velocity profile had little effect on the flow compared with the effect of changing the curvature of the model. From our study, it is evident that the effect of changing the shape of the primary flow inlet velocity profile is not felt significantly beyond 1.75*D*, with *D* being the aortic root diameter. However, upstream of 1*D*, the shape of the axial flow can lead to as much as $18\%$ variability in terms of time-averaged wall shear stress. Adding secondary flow on top of parabolic axial flow also results in significant variability in wall shear stress upstream of 1*D*, as high as $26\%$ during diastole. Consequently, if accurate temporal modeling closer to the inlet and the aortic arch is desired, our results emphasize the need to model patient-specific inlet velocity conditions including secondary flow.

### Effect of outlet boundary conditions

Table 5 illustrates the differences in wall shear stress magnitude between the three-element Windkessel model, the two-element Windkessel model, and the prescribed percentage outflow boundary conditions. All three of these cases had identical parabolic inlet axial velocity conditions and no secondary flow at the inlet. The data show no significant difference in wall shear stress between the two-element Windkessel and the three-element Windkessel conditions. However, the two-element and the three-element models vary as much as about $18~\%$ from the case with an outflow boundary condition. Comparing these results with the magnitude of variations from other factors suggests that outlet boundary conditions are a significant contributor to uncertainty in the solution.

**Fig. 9:**
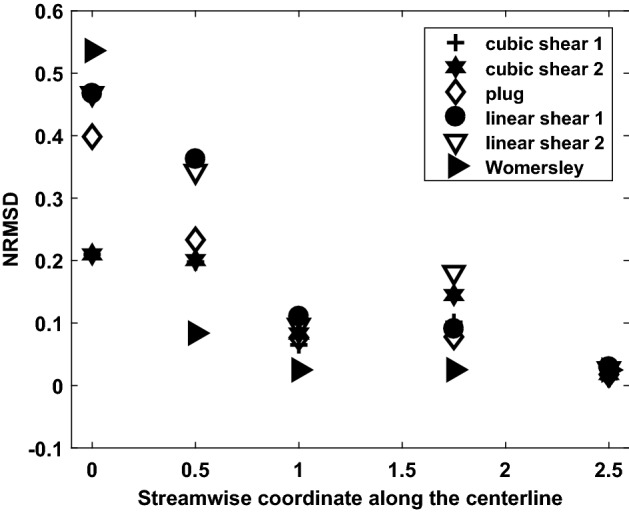
Normalized root-mean-square deviation (*NRMSD*) of axial velocity magnitude compared with outflow outlet boundary condition at various locations along the streamwise coordinate during peak systole; the streamwise coordinate is measured in multiples of *D*, the vessel’s inlet diameter. These simulations had parabolic inlet boundary condition with no secondary flow

Figure 9 shows the differences between the Windkessel boundary conditions and the outflow condition using normalized root-mean-square deviation (*NRMSD*) of axial velocity magnitude as described in Eq. 5, integrated over cross-sectional slices at the coordinates indicated. A general increase in *NRMSD* is observed with increasing streamwise coordinate, although there is a slight decrease at 4.5*D* relative to that observed at 3.5*D*. Furthermore, variation in *NRMSD* beyond 3.5*D* is constant within $2.5\%$ for both the Windkessel boundary conditions examined. The fact that *NRMSD* is highest near the outlet is expected when comparing cases that vary outlet conditions. However, it is notable that whereas *NRMSD* decayed nearly to zero for all inlet conditions by 2.5 diameters from the inlet, *NRMSD* remained high more than 5 diameters proximal to the outlet. This suggests that the choice of outlet condition has a noticeable effect on a larger percentage of the solution domain than the choice of inlet condition.

## Conclusions and summary

This work investigated the variation introduced into a simulation of aortic blood flow by choice of inlet and outlet boundary conditions.

Inlet plug flow, parabolic flow, linear shear flows, skewed cubic flows, and Womersley flow were simulated and the resulting flow solutions were compared to study the effect of inlet conditions. Parabolic flow with and without secondary flow at the inlet was also studied. All other parameters were identical among these simulations. While the parabolic inlet condition without secondary flow has the lowest time-averaged wall shear stress, linear shear flow and plug flow have the highest time-averaged wall shear stress, about $8\%$ higher than parabolic inlet condition without secondary flow. The axial and in-plane velocities for the different flow solutions are considerably different across data slices extracted at 0.5*D* and 1*D* from the inlet, where *D* is the inlet diameter. Data slices at 1.75*D* and 2.5*D* are qualitatively similar but there are minor differences between secondary flows at 1.75*D*. Normalized root-mean-square deviation (*NRMSD*) evaluated between the parabolic inlet condition without secondary flow and other axial velocity boundary conditions generally decreases along the streamwise coordinate and is less than 0.03 at 2.5*D* for all cases. These statistics show that the effect of inlet conditions becomes less pronounced as the streamwise coordinate increases. Adding secondary inlet flow to parabolic axial flow results in a slight variation of about $4\%$ in terms of the time-averaged wall shear stress. However, between the inlet and a streamwise coordinate of 1*D*, there are larger differences. This is especially noticeable during diastole when shear stress magnitude differences integrated up to 1D are as high as $26\%$.

Outlet conditions prescribing a zero-diffusion flux with specified mass flow rate (*ANSYS® Academic Research [Fluent], release 16.2, outflow boundary conditions, ANSYS, Inc.*), two-element Windkessel, and the three-element Windkessel conditions were investigated. Both the two-element and the three-element Windkessel models don’t vary much near the inlet as seen from the time-averaged wall shear stress variations. For instance, both the two-element and the three-element models differ from the outflow boundary condition by 0.3544 and $0.3571\%$ respectively in terms of time-averaged wall shear stress integrated up to 1*D*. However, in terms of time-averaged wall shear stress integrated throughout the model, they differ from the outflow boundary condition by as much as about $18\%$. Normalized root-mean-square deviation (*NRMSD*) evaluated between the outflow boundary condition and the Windkessel models generally increases along the streamwise coordinate. However, beyond 3.5*D* *NRMSD* varies by less than $2.5\%$ along the streamwise coordinate. These statistics indicate that *NRMSD* remains constant for more than 5 diameters proximal to the outlet and that the effect of outlet conditions are more pronounced as the streamwise coordinate increases.

Based on the current results along with other studies on the subject [70, 89, 90], it is reasonable to conclude that inlet conditions, including both primary and secondary velocity profile shape, significantly affect the solution up to about two inlet diameters distal to the inlet. Similarly, the type of outlet condition chosen affects the solution significantly up to five inlet diameters proximal to the outlet. This suggests that the outlet boundary conditions influence a larger percent of the solution domain. The amount of variation observed between the various flow cases in this study can be interpreted as a lower bound on the error that can be expected in aortic flow simulations that do not use patient-specific boundary conditions. Although this study is limited to one healthy model, the underlying mechanisms of flow over the curvature of the vessel and the effect of branches would likely render qualitatively similar results in other subject-specific models. Nevertheless, studying more subject-specific models along with corresponding physiologically realistic inlet velocity boundary conditions to verify our conclusions is of interest for future work.

## List of symbols

### Symbols

D: diameter of aorta at the inlet; $\overrightarrow{U}$: axial velocity vector.

### Greek letters

$$\tau _{}$$: shear stress.

### Non-dimensional numbers

$$\mathbf {Re}$$: Reynolds number.

### Subscripts or superscripts

*w*: wall.

### Acronyms and abbreviations

*NRMSD*: normalized root-mean-square deviation; *WSS*: wall shear stress; *TAWSS*: time-averaged wall shear stress.
